# Acute and Long-Term Effects of Attentional Focus Strategies on Muscular Strength: A Meta-Analysis

**DOI:** 10.3390/sports9110153

**Published:** 2021-11-12

**Authors:** Jozo Grgic, Ivan Mikulic, Pavle Mikulic

**Affiliations:** 1 Institute for Health and Sport, Victoria University, Melbourne, VIC 3011, Australia; 2 Faculty of Kinesiology, University of Zagreb, 10000 Zagreb, Croatia; ivan.mikulic@kif.unizg.hr (I.M.); pavle.mikulic@kif.unizg.hr (P.M.)

**Keywords:** muscle strength, resistance training, data analysis, attention

## Abstract

This review aimed to perform a meta-analysis examining the following: (a) acute effects of adopting an internal focus vs. external focus of attention on muscular strength; and (b) long-term effects of adopting an internal focus vs. external focus of attention during resistance training on gains in muscular strength. We searched through five databases to find eligible studies. Random-effects meta-analyses of standardized mean differences were conducted to analyze the data. Ten studies were included. In the meta-analysis for the acute effects, there was a significant positive effect of external focus on muscular strength (standardized mean difference: 0.34; 95% confidence interval: 0.22, 0.46). In the meta-analysis for the long-term effects, there was no significant difference between training with an internal focus and external focus on muscular strength gains (standardized mean difference: 0.32; 95% confidence interval: –0.08, 0.73). In the subgroup analysis for lower-body exercises, we found a significant positive effect of training with an external focus on muscular strength gains (standardized mean difference: 0.47; 95% confidence interval: 0.07, 0.87). In summary, our findings indicate an acute increase in muscular strength when utilizing an external focus of attention. When applied over the long-term, using an external focus of attention may also enhance resistance training-induced gains in lower-body muscular strength.

## 1. Introduction

The importance of attentional focus on motor learning is well-established [1]. Most commonly, studies compare the effects of internal focus vs. external focus of attention [1]. Internal focus involves focusing on bodily movements, whereas external focus consists of focusing on an external object related to the task [1]. Wulf et al. [2] published a seminal study that compared the effects of internal focus vs. external focus on motor learning. This study found that adopting an external focus enhanced motor learning of slalom-type movements on a ski-simulator. Since this work, many other studies have been published on this topic. These studies have generally demonstrated that adopting an external focus of attention is beneficial for the performance of tasks in different sports such as golf, rowing, and basketball [3,4,5]. Research in the field also explored the effects of attentional focus strategies on movement coordination and landing biomechanics in athletes with anterior cruciate ligament reconstruction [6,7].

Studies have also compared the effects of external vs. internal focus on resistance exercise performance [8,9,10,11]. The majority of studies performed in this area evaluated the effects of these strategies on muscle activation using surface electromyography (EMG) [8,9,11,12]. Besides EMG, studies have evaluated the effects of internal vs. external focus on muscular strength, with equivocal findings. For example, Halperin et al. [13] compared the effects of external vs. internal focus on force production in the isometric mid-thigh pull exercise. This study observed that adopting an external focus of attention resulted in a 9% higher peak force production. However, Marchant and Greig [14] did not replicate these findings, as in their study, there was no significant difference in isokinetic peak torque between internal and external focus. Therefore, while several studies explored the effect of different attentional focus strategies on muscular strength, there is still no consensus on this topic.

Besides acute effects, studies have also conducted training interventions in which participants received either external focus or internal focus cues during each exercise session [15,16]. Some of these studies reported greater gains in muscular strength when adopting an external focus of attention, whereas others found no significant differences between these two motor learning strategies [15,16]. Given the inconsistent evidence on the topic, this review aimed to perform a meta-analysis examining: (a) acute effects of adopting an internal focus vs. external focus of attention on muscular strength; and (b) long-term effects of adopting an internal focus vs. external focus of attention during resistance training on gains in muscular strength. Such an analysis would be of practical relevance given the importance of muscular strength for athletic performance and activities of daily living [17,18].

## 2. Materials and Methods

### 2.1. Search Strategy

For this review, we followed the Preferred Reporting Items for Systematic Reviews and Meta-Analyses (PRISMA) guidelines [19]. We searched through Open Access Theses and Dissertations, Networked Digital Library of Theses and Dissertations, PubMed/MEDLINE, Scopus, and Web of Science. The following search syntax was utilized: (“external focus” OR “attentional focus” OR “internal focus”) AND (strength OR “one-repetition maximum” OR 1RM OR 1-RM OR MVC OR “maximal voluntary contraction” OR “torque” OR “force production” OR “handgrip”). The search results from each database were downloaded and subsequently filtered in EndNote software (X8; Clarivate Analytics, New York, USA). After completing the primary search on 16 December 2020, two secondary searches were performed. We first screened the reference list from all included studies and then conducted forward citation tracking, which consists of searching through the papers citing the included studies using Google Scholar and Scopus.

### 2.2. Inclusion Criteria

We included studies that satisfied the following criteria: (1) written in English; (2) explored the acute or long-term effect of external focus vs. internal focus on attention on muscular strength; (3) utilized a crossover study design (for studies examining acute effects) or a between-group design (for studies exploring long-term effects); and (4) presented mean ± standard deviation (SD) data from the muscular strength test. All studies that did not satisfy these criteria were excluded from the review. The most common reason for excluding studies was that they did not evaluate muscular strength. 

### 2.3. Data Extraction 

From each included study, we extracted the following data on a coding sheet using Microsoft Excel software (Microsoft Corporation, Redmond, WA, USA): (1) lead author name and year of study publication; (2) participants characteristics; (3) cues provided for the external focus and internal focus; (4) test(s) used to evaluate muscular strength; and (5) main study findings.

### 2.4. Methodological Quality

We assessed the methodological quality of studies included in the analysis using the PEDro checklist [20]. This checklist contains 11 items, which evaluate various methodological aspects (e.g., inclusion criteria, randomization, blinding, data reporting). Per the PEDro assessment guidelines, items are scored with “1” (criterion is satisfied) or “0” (criterion is not satisfied). While there are 11 items on the list, the maximum possible score is 10, given that the first item is not included in the summary score. We classified studies as excellent (9–10 points), good (6–8 points), fair (4–5 points), and poor (≤3 points) methodological quality [21,22].

### 2.5. Statistical Analysis

Meta-analyses were performed using standardized mean differences (SMDs) in the random-effects model. The muscular strength data were converted to SMDs with their 95% confidence intervals (CI). For the acute effects, the muscular strength mean ± SD data from the external focus and internal focus trials, total sample size, and inter-trial correlation are used to calculate SMDs. Given that the studies did not present inter-trial correlation, we estimated correlation values using the recommendations in the Cochrane Handbook [23]. In the meta-analysis for long-term effects, SMDs were calculated using pre-intervention and post-intervention mean ± SD data from the external focus and internal focus groups and their respective sample sizes. SMDs were calculated as pre-post intervention mean change, divided by the pooled SD. For studies that presented multiple related outcomes, SMDs and variances were calculated for each outcome. Then, average values were utilized in the meta-analysis. In the meta-analysis for the long-term effects, we conducted a subgroup analysis where we analyzed only gains in lower-body muscular strength. To interpret SMD values, we used the following thresholds: “small” (0.20–0.49), “medium” (0.50–0.79), and “large” (≥0.80) [24]. We assessed heterogeneity using *I^2^*, which was interpreted as low (*I^2^* < 50%), moderate (*I^2^ =* 50–75%), and high heterogeneity (*I^2^* >75%). The statistical significance threshold was set at *p* < 0.05. All analyses were performed using the Comprehensive Meta-analysis software, version 2 (Biostat Inc., Englewood, NJ, USA).

## 3. Results

### 3.1. Search Results

**Figure 1:**
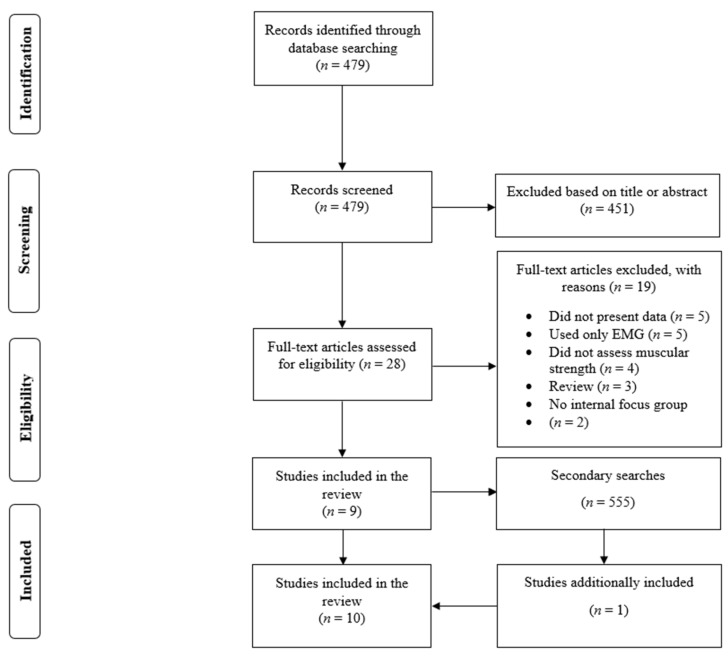
Depiction of the search process.

In the primary search, there was a total of 479 search results (Figure 1). We excluded 451 search results after reading the title or abstract. Therefore, 28 full-text papers were read. Nineteen studies were excluded after reading the full texts, and a total of nine studies were included in the review [13,14,15,16,25,26,27,28,29]. There were 555 search results in the secondary searches, and one additional study was included in the review [30].

### 3.2. Summary of Studies

Seven studies compared the acute effects of external vs. internal focus on muscular strength (Table 1). The sample sizes in these studies ranged from 11 to 30 participants. Studies used different strength tests, such as the isometric mid-thigh pull, handgrip strength, isometric elbow flexion, squat, and deadlift. Three studies compared the long-term effects of external vs. internal focus on muscular strength [15,16,30]. The sample sizes in these three studies ranged from 20 to 44 participants. Muscular strength was evaluated using the squat, deadlift, knee extension, or elbow flexion. The duration of the interventions was from 6 to 12 weeks. Specific cues provided to the participants are summarized in Table 1.

### 3.3. Methodological Quality

Studies scored from 5 to 7 points on the PEDro checklist (median: 6 points). Seven studies were classified as having good methodological quality, while three studies were classified as being of fair methodological quality (Table 1).

### 3.4. Meta-Analysis Results

**Figure 2:**
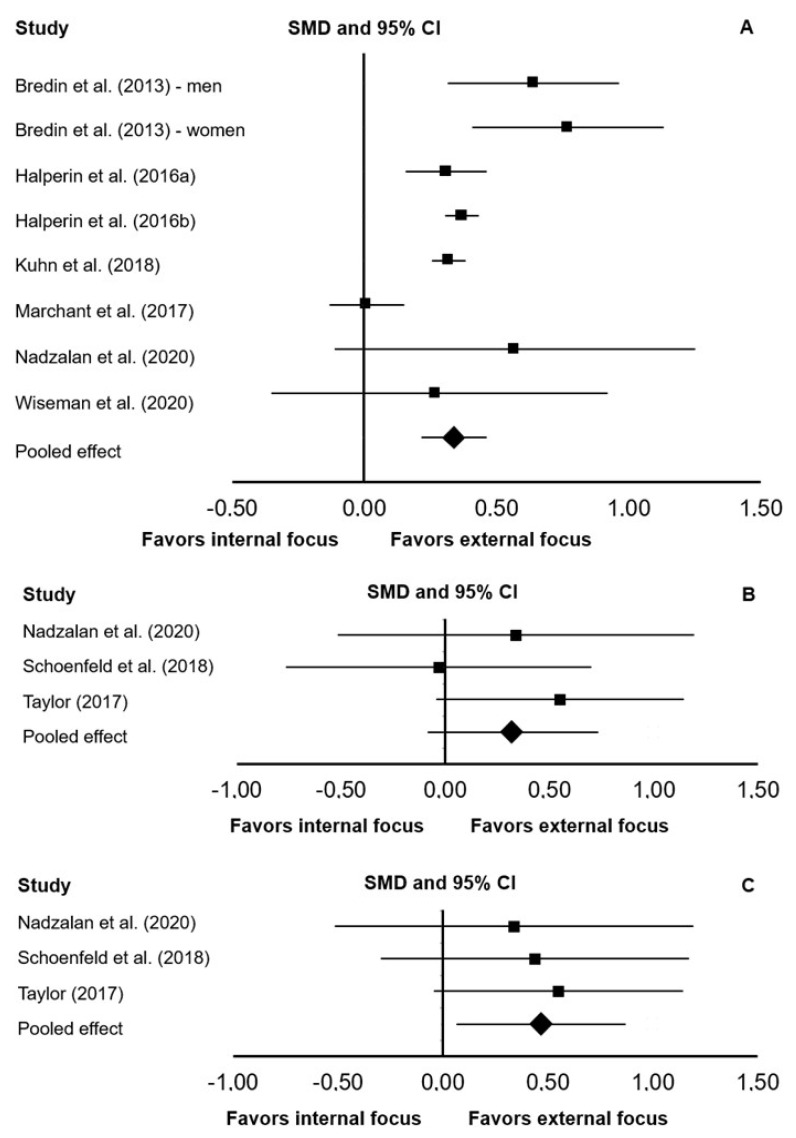
Results from the meta-analysis that explored the acute effects of internal focus vs. external focus on muscular strength (**A**), long-term effects of an internal focus vs. external focus on muscular strength (**B**), long-term effects of an internal focus vs. external focus on lower-body muscular strength (**C**). The data are presented as squares, which represent standardized mean differences (SMD) and whiskers, which are 95% confidence intervals (CIs). The diamond represents the pooled effect [13,14,15,16,25,26,27,28,29,30].

In the meta-analysis for the acute effects, there was a significant positive effect of external focus on muscular strength (SMD: 0.34; 95% CI: 0.22, 0.46; *p* < 0.001; *I^2^* = 40%; Figure 2). In the meta-analysis for the long-term effects, there was no significant difference between training with an internal focus and external focus on gains in muscular strength (SMD: 0.32; 95% CI: –0.08, 0.73; *p* = 0.113; *I^2^* = 0%). In the subgroup analysis that considered only lower-body exercises, there was a significant positive effect of training with an external focus on gains in muscular strength (SMD: 0.47; 95% CI: 0.07, 0.87; *p* = 0.023; *I^2^* = 0%).

## 4. Discussion

In this meta-analysis, we found an acute increase in muscular strength when adopting an external focus of attention. There were no significant differences in the main meta-analysis that compared the effects of training with an internal focus vs. external focus of attention on muscular strength gains. However, in a subgroup analysis that considered only lower-body strength gains, we found a significant positive effect of training with an external focus. In summary, our findings indicate an acute increase in muscular strength when utilizing an external focus of attention. When applied over the long-term, using an external focus of attention may also enhance resistance training-induced gains in lower-body muscular strength.

Our results support previous research showing that external focus enhances motor performance. In a comprehensive review, Wulf [1] demonstrated that adopting an external focus of attention enhances accuracy, balance, jumping performance, and other sport-specific exercise outcomes. The data presented in this meta-analysis is in accord with the *constrained action hypothesis* [1,31]. The *constrained action hypothesis* suggests that adopting an internal focus of attention may lead the individual to focus only on one component of the movement (e.g., only on one muscle activated during the tests). Indeed, in one of the included studies [14], the authors provided the following internal focus cue before isokinetic knee extension: “Contract the vastus medialis oblique whilst generating maximal effort.” Providing such cues may constrain the motor system and result in individuals focusing on only one component responsible for completing the task. This might hinder performance, given that the movement is often achieved by an integration of many muscles. The *constrained action hypothesis* suggests that providing external focus cues may allow the execution of the tasks without omitting any of the contributors, ultimately resulting in better performance [1,31]. Adopting an external focus may also result in the following: (a) more effective contraction strategies (i.e., reduced co-activation and EMG amplitudes); and (b) reduced rating of perceived exertion [27,29,30]. All these factors are believed to contribute to improved performance with an external focus of attention.

From a practical perspective, our results are likely most relevant in two areas. Firstly, the results presented herein may be of substantial practical importance for athletes competing in strength-based sports such as powerlifting and weightlifting. Our findings suggest that adopting an external focus of attention may enhance muscular strength and, therefore, directly impact performance and success in these sports. Secondly, our results highlight the importance of cue standardization when conducting muscular strength tests [32]. Strength testing is commonly used to evaluate the efficacy of training programs as well as for exercise prescription [33]. Theoretically, if the assessors provide different cues during the test and retest sessions, this may impact muscular strength performance and subsequently influence the correct interpretation of the data. For the purpose of strength testing, our results suggest that providing cues that impact the focus of attention should be either standardized or avoided altogether.

While there was no significant difference in the main meta-analysis for the long-term effects of attentional focus strategies, we found that resistance training with an external focus results in significantly greater lower-body muscular strength gains. These results suggest that the acute effects, when applied in each training session, may also impact lower-body strength gains over the long-term. While the topic of internal versus external focus of attention during training intervention requires much more research, our results preliminarily support the positive effects of an external focus on motor performance. In line with the data presented herein, previous research also showed a benefit toward an external focus of attention during nine weeks of plyometric training on vertical jump performance [34]. Furthermore, one study used a seven-day balance training intervention, where one group performed 30 balance trials per day with internal focus cues and one group with external focus cues [35]. Post-intervention, this study demonstrated a benefit for performance in a balance test in the group training with an external focus [35].

All three studies that conducted resistance training interventions assessed lower-body strength using exercises such as the deadlift, squat, and knee extension. Out of these three studies, only one used an upper-body test of strength (i.e., isometric elbow flexion) [16]. Based on this comparison of exercises used for the strength test, it might be that the effects of adopting an external focus of attention are greater in lower body vs. upper body or in complex vs. simple exercises. The latter idea has more support in the literature, as Wulf [36] suggested that external focus benefits are larger in more complex movements that require a greater level of multi-muscle and multi-joint coordination. Therefore, using an external focus may impact strength gains to a larger extent in complex vs. simple exercises. Nevertheless, future research is still needed to further investigate the effect of resistance training with an internal or external focus in various upper- and lower-body strength tests.

Based on the PEDro checklist, the included studies were of fair or good methodological quality. Still, it should be considered that only two of the included studies incorporated blinding as a part of their design [13,30]. These two studies blinded the included participants to the aims of the study. However, none of the included studies reported blinding of the assessors. While acknowledging the logistical challenges that researchers face, future studies on the topic would benefit from incorporating a double-blind design. Additionally, one of the studies that investigated the long-term effects did not report participants’ compliance with the training programs [15]. This should be mentioned, given that exercising with an external focus may reduce perceived exertion and might be more enjoyable, possibly impacting adherence [30]. These limitations should be addressed in future studies. 

There are several limitations of this review that should be acknowledged. Specifically, there were no significant differences between the conditions in the main meta-analysis that explored the long-term effects of external vs. internal focus. The lack of statistically significant findings in this analysis might be because only three studies were included, highlighting the need for future studies. Additionally, while our findings suggest a benefit of using an external focus of attention for muscular strength, such recommendations should not be generalized to other resistance training adaptations such as muscular hypertrophy. To our knowledge, only one study evaluated the effects of external vs. internal focus of attention on muscular hypertrophy and actually reported a benefit toward training with an internal focus of attention [16]. Therefore, future studies are needed to explore the effects of attentional focus strategies on muscular hypertrophy.

## 5. Conclusions

Compared to an internal focus, we found an acute increase in muscular strength when adopting an external focus of attention. There were no significant differences in the main meta-analysis that compared the effects of training with an internal focus vs. external focus of attention on muscular strength gains. However, in a subgroup analysis that considered only lower-body strength gains, we found a significant positive effect of training with an external focus. In summary, our findings indicate an acute increase in muscular strength when utilizing an external focus of attention. When applied over the long-term, using an external focus of attention may also enhance resistance training-induced gains in lower-body muscular strength.

## Figures and Tables

**Table 1:** Summary of the included studies.

Reference	Participants	External Focus Instructions	Internal Focus Instructions	Exercise Test	Training Intervention	Pedro Score
Bredin et al. (2013) [25]	8 young men and 8 young women	Concentrate on the wall marker during the test	Concentrate specifically on the fingers	Handgrip strength	n/a	6
Halperin et al. (2016a) [13]	18 trained athletes (10 men and 8 women)	“Focus on pushing the ground as hard and as fast as you possibly can.”	“Focus on contracting your leg muscles as hard and as fast as you possibly can.”	Isometric mid-thigh pull	n/a	7
Halperin et al. (2016b) [26]	28 resistance-trained participants (14 men and 14 women)	“Attempt to produce as much force as you possibly can while focusing on pulling the strap as hard and as fast as you can.”	“Attempt to produce as much force as you possibly can while focusing on contracting your arm muscles as hard and as fast as you can.”	Elbow flexion MVC	n/a	6
Kuhn et al. (2018) [27]	14 participants (11 men and 3 women)	“Exert pressure on the force transducer so that the moving line increases as fast as possible to the maximum after the tone.”	“Contract your finger flexor muscles so that the moving line increases as fast as possible to the maximum after the tone.”	Index finger flexion MVC	n/a	6
Marchant et al. (2017) [14]	20 resistance-trained participants (16 men and 4 women)	“Try to exert maximal effort during the movement whilst focusing on pushing against the pad.”	“Contract the vastus medialis oblique whilst generating maximal effort.”	Isokinetic leg extension	n/a	5
Nadzalan et al. (2019) [15]	20 resistance-trained men	Deadlift: “Focus your attention on pulling the bar up.” Squat: “Focus on moving and exerting force through and against the barbell.”	Deadlift: “Focus your attention on extending your knees and hips.” Squat: “Focus on moving and exerting force with your legs.”	Squat and deadlift 1RM	6 weeks	5
Nadzalan et al. (2020) [28]	30 resistance-trained men	Deadlift: “Focus your attention on pulling the bar up.” Squat: “Focus on moving and exerting force through and against the barbell.”	Deadlift: “Focus your attention on extending your knees and hips.” Squat: “Focus on moving and exerting force with your legs.”	Squat and deadlift 10RM	n/a	5
Schoenfeld et al. (2018) [16]	27 untrained men	“Get the weight up!”	“Squeeze the muscle!”	Knee extension and elbow flexion MVC	8 weeks	6
Taylor (2017) [30]	44 male university team sport athletes	Squat: “Focus on driving the bar to the ceiling as explosively as possible.” Deadlift: “Focus on pushing the ground away as fast as possible.”	Squat: “Focus on extending at your knees as rapidly as possible.” Deadlift: “Focus on extending at your hips as rapidly as possible.”	Squat and deadlift 1RM	12 weeks	7
Wiseman et al. (2020) [29]	11 resistance-trained men	“Focus on pulling up on the handle as hard and as quickly as you possibly can.”	“Focus on contracting your biceps as hard and as quickly as you possibly can.”	Elbow flexion MVC	n/a	6

MVC: maximum voluntary contraction; RM: repetition maximum. n/a: not applicable

## Data Availability

Data used for the meta-analysis are available on request from the corresponding author.
